# Primordial Germ Cell Specification from Embryonic Stem Cells

**DOI:** 10.1371/journal.pone.0004013

**Published:** 2008-12-24

**Authors:** Wei Wei, Tingting Qing, Xin Ye, Haisong Liu, Donghui Zhang, Weifeng Yang, Hongkui Deng

**Affiliations:** 1 Laboratory of Chemical Genomics, School of Chemical Biology and Biothechnology, Shenzhen Graduate School of Peking University, Shenzhen, China; 2 Laboratory of Stem Cell and Generative Biology, College of Life Sciences, Peking University, Beijing, China; 3 INSERM/UEVE U861 I-Stem, AFM, Evry, France; 4 Cardiovascular Research Laboratory, University of British Columbia, St Paul's Hospital, Vancouver, British Columbia, Canada; Ecole Normale Supérieure de Lyon, France

## Abstract

**Background:**

Primordial germ cell (PGC) specification is the first crucial step in germ line development. However, owing to significant challenges regarding the *in vivo* system, such as the complex cellular environment and potential problems with embryo manipulation, it is desirable to generate embryonic stem (ES) cells that are capable of overcoming these aforementioned limitations in order to provide a potential *in vitro* model to recapitulate the developmental processes *in vivo*.

**Methodology and Principal Findings:**

Here, we studied the detailed process of PGC specification from *stella*-GFP ES cells. We first observed the heterogeneous expression of *stella* in ES cells. However, neither Stella-positive ES cells nor Stella-negative ES cells shared a similar gene expression pattern with either PGCs or PGC precursors. Second, we derived PGCs from ES cells using two differentiation methods, namely the attachment culture technique and the embryoid body (EB) method. Compared with PGCs derived via the attachment culture technique, PGCs derived via the EB method that had undergone the sequential erasure of *Peg3* followed by *Igf2r* resulted in a cell line in which the expression dynamics of *T*, *Fgf8* and *Sox17*, in addition to the expression of the epiblast markers, were more similar to the *in vivo* expression, thus demonstrating that the process of PGC derivation was more faithfully recapitulated using the EB method. Furthermore, we developed an *in vitro* model of PGC specification in a completely chemically defined medium (CDM) that indicated that BMP4 and Wnt3a promoted PGC derivation, whereas BMP8b and activinA had no observable effect on PGC derivation.

**Conclusions and Significance:**

The *in vitro* model we have established can recapitulate the developmental processes *in vivo* and provides new insights into the mechanism of PGC specification.

## Introduction

The investigation of primordial germ cell (PGC) specification is the first essential step in the process of elucidating the mechanisms involved in the development of a germ cell lineage. However, significant difficulties exist with regard to research into the process of PGC specification *in vivo*. First, the complex *in vivo* environment of the cell has led to controversies over the mechanism of PGC development [1], [2]. In addition, PGCs are difficult to study because they are limited in number, deeply embedded within the embryo, and are known to migrate during development [3]–[5], which mitigates the degree to which they can be effectively studied. Moreover, large-scale screens of potential inducers of the PGC specification process are difficult to implement. Hence, embryonic stem (ES) cells, which have overcome these aforementioned difficulties, provide promising candidates to recapitulate the developmental process *in vitro* and thus serve as a model to complement studies *in vivo*.

Previous studies have demonstrated that ES cells are capable of differentiating into germ cells in either the attachment culture technique or the EB method [6]–[12]. Nayernia *et al.* showed that live-birth mice could be obtained from spermatozoa that were completely derived *in vitro* from ES cells [10]. In addition, oocytes were derived from *gcOct4*-GFP ES cells in a study reported by Hübner *et al.* [6]. Although such reports have indicated the ability to successfully study germ cell development *in vitro*, the process of PGC specification is poorly understood. First, the parental imprints—which must be erased and reset during gametogenesis, reflecting the sex of the individual, and must be maintained in somatic cells after fertilization [13]—have been examined only in derived embryonic germ cells [8]. However, no derived PGCs have been tested for this property [6]–[12]. Second, the BMP pathway, which is confirmed to induce PGC specification of the proximal epiblast *in vivo* [14], has proven to function in an obscure fashion [7]. PGCs were rapidly derived from ES cells by co-aggregating the PGCs with BMP4 producing cells, whereas neither the direct addition of BMP4 to the medium nor the preparation of BMP4-producing feeder cells could obtain this effect. Moreover, the fundamental question of how PGCs are derived *in vitro* remains to be answered, although three current hypotheses exist. These hypotheses include the ideas that ES cells may already include PGCs, that ES cells may directly differentiate into PGCs, and, finally, that PGCs develop through an intermediate state, such as an epiblast-like stage [15].

Due to the fact that a significant number of markers are shared between PGCs and ES cells, the careful study of PGC specification *in vitro* is difficult. Pluripotent markers, such as Oct4 and SSEA1, are both expressed in ES cells and PGCs. In addition, PGC markers, such as *Blimp1*, *Mvh*, *Fragilis* and *stella* and even germ cell specific markers, such as *Piwil2*, *Rnh2*, *Tdrd1* and *Tex14*, are detected in ES cells [8], [12], [16]. Recently a systematic analysis of single cell expression has revealed the gene expression dynamics in germ-line cells during PGC specification *in vivo* [17] and indicated differential expression patterns between ES cells and PGCs, such as their expression of *Eras*, *T*, and *Fgf8*. In addition, the gene expression profiles in common ancestors of the nascent germ cells and their somatic neighbors demonstrate that the most specific gene for the germ cell is *stella* [18], indicating an excellent sorting marker for studying PGC specification *in vitro*.

In this study, we aim to elucidate PGC specification using an ES cell line expressing *stella*-GFP derived from a *stella*-GFP BAC transgene that lacks any ectopic expression [19]. Here, we have shown that subpopulations of the *stella*-GFP ES cells were heterogeneous in terms of *stella* expression, but none of these subpopulations shared similar expression patterns with either PGC precursors or PGCs prior to E7.75. In addition, analysis of the dynamic gene expression patterns of the derived PGCs using the attachment culture technique and the EB method indicated that the process of PGC specification was more faithfully recapitulated using the EB method than with the former technique. Moreover, we have developed an *in vitro* model for PGC specification providing a convenient strategy to screen new factors or small molecules that will potentially lead to the elucidation of the mechanism for PGC specification.

## Results

### ES cells may not contain PGC precursors or PGCs

**Figure 1:**
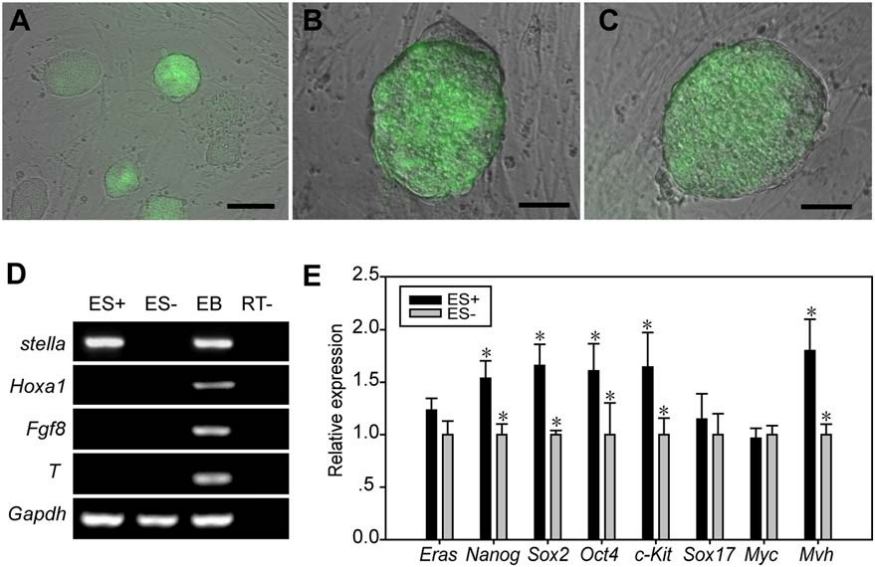
Heterogeneity of *stella*-GFP ES cells. (A, B, C) A merged view of the phase contrast images and fluorescence images of *stella*-GFP expression (green). (A) *stella*-GFP ES cells in an ES medium; bar = 100 µm. After the *stella*-GFP ES cells were sorted by *stella* expression, we found that single GFP-positive ES cells formed clones containing both GFP-negative and GFP-positive ES cells (B) and that single GFP-negative ES cells also generated such clones (C); bar = 50 µm. The ratio of clones including the GFP-positive cell in the GFP-negative and GFP-positive descendents was 78.8±3% and 82.9±5%, respectively. Furthermore, the ratio of GFP-positive cells in GFP-negative descendents was roughly 15%, which was the same as in the GFP-positive descendents and in the unsorted *stella*-GFP ES cells. (D) RT-PCR gene expression analysis in GFP-positive ES cells (ES+) and GFP-negative ES cells (ES−). *Hoxa1*, a somatic marker [17], was not expressed in ES+ or ES− cells. *Fgf8* and *T*, expressed in PGCs prior to E7.75 [17], were also undetectable in both ES+ and ES− cells. (E) Quantitative RT-PCR (Q-PCR) gene expression analysis in ES+ and ES− cells. The relative expression of each gene in differentiated cultures was normalized by its expression in ES− cells after normalization to *Gapdh*. * P<0.05. *Eras*, which is not expressed in PGCs, is a specific marker for ES cells [17]. *Eras* was expressed at similar levels between ES+ and ES− cells. The pluripotent markers *Nanog*, *Sox2*, *Oct4* and *c-kit* were more highly expressed in ES+ than in ES− cells. *Sox17*, which is expressed in the epiblast and transiently upregulated in PGCs during PGC development [17], was expressed at extremely low levels in ES+ and ES− cells. *Myc*, a pluripotent marker, which is repressed during PGC development [17], showed similar expression in ES+ and ES− cells. *Mvh*, a marker of post-migratory PGCs [7], which is also expressed in ES cells [16], was more highly expressed in ES+ than in ES− cells.

It has been proposed that ES cells may already include PGCs or PGC precursors [15]. To test this hypothesis, the properties of *stella*-GFP ES cells were investigated. We found that the *stella*-GFP ES cells did not ubiquitously express *stella* (Fig. 1A), and the two subpopulations in terms of stella expression were interchangeable (Figs. 1B and C). To explore whether GFP-positive or GFP-negative ES (ES+, ES−, respectively) cells possessed similar expression patterns for PGCs or its precursors, the expression patterns of PGC-related genes were compared. The genes expressed in different stages of the PGC precursors and PGCs prior to E8.25 are summarized in Table 1 [17]. The expression of *stella* in ES+ and ES− cells verified the quality of the FACS result (Fig. 1D). The differentially expressed genes in the ES cells and in the different stages of PGCs were clearly *Eras*, *Myc*, *Sox17*, *Fgf8*, *T* and *stella* (Table 1, Figs. 1D and E). The high expression of *Eras* and the undetectable expression of both *Fgf8* and *T* in ES+ and ES− cells (Figs. 1D and E) indicated that ES cells did not contain PGCs prior to E7.75. However, the expression patterns in ES+ could not completely exclude the existence of E8.25 PGCs (Table 1). Furthermore, *Mvh*, a marker for post-migratory PGCs [7] that is also known to be expressed in ES cells [9], [12], [16], was shown to be expressed at higher levels in ES+ than in ES− cells (Fig. 1E). Thus, the expression of *Mvh* may be a property of ES cells. Taken together, these results indicate that ES− cells may not contain either PGCs or PGC precursors; whereas ES+ may not include cells equivalent to PGCs prior to E7.75.

**Table 1:** Summary of gene expression in ES cells and different stages of PGCs.

	*Eras*	*Hoxa1*	*Nanog*	*Oct4*	*Myc*	*c-Kit*	*Sox2*	*Sox17*	*Fgf8*	*T*	*stella*
E6.75*	−	−	+	+	+	+/−	+/−	−	+	+	−
E7.25	−	−	+	+	−	+	+	+	+	+	+
E7.75	−	−	+	+	−	+	+	+/−	+	+	+
E8.25	−	−	+	+	−	+	+	−	−	−	+
ES+	+	−	+	+	+	+	+	+/−	−	−	+
ES−	+	−	+	+	+	+	+	+/−	−	−	−

* E = embryonic day.

### Differentiation methods affect the yield of PGCs from ES cells

**Figure 2:**
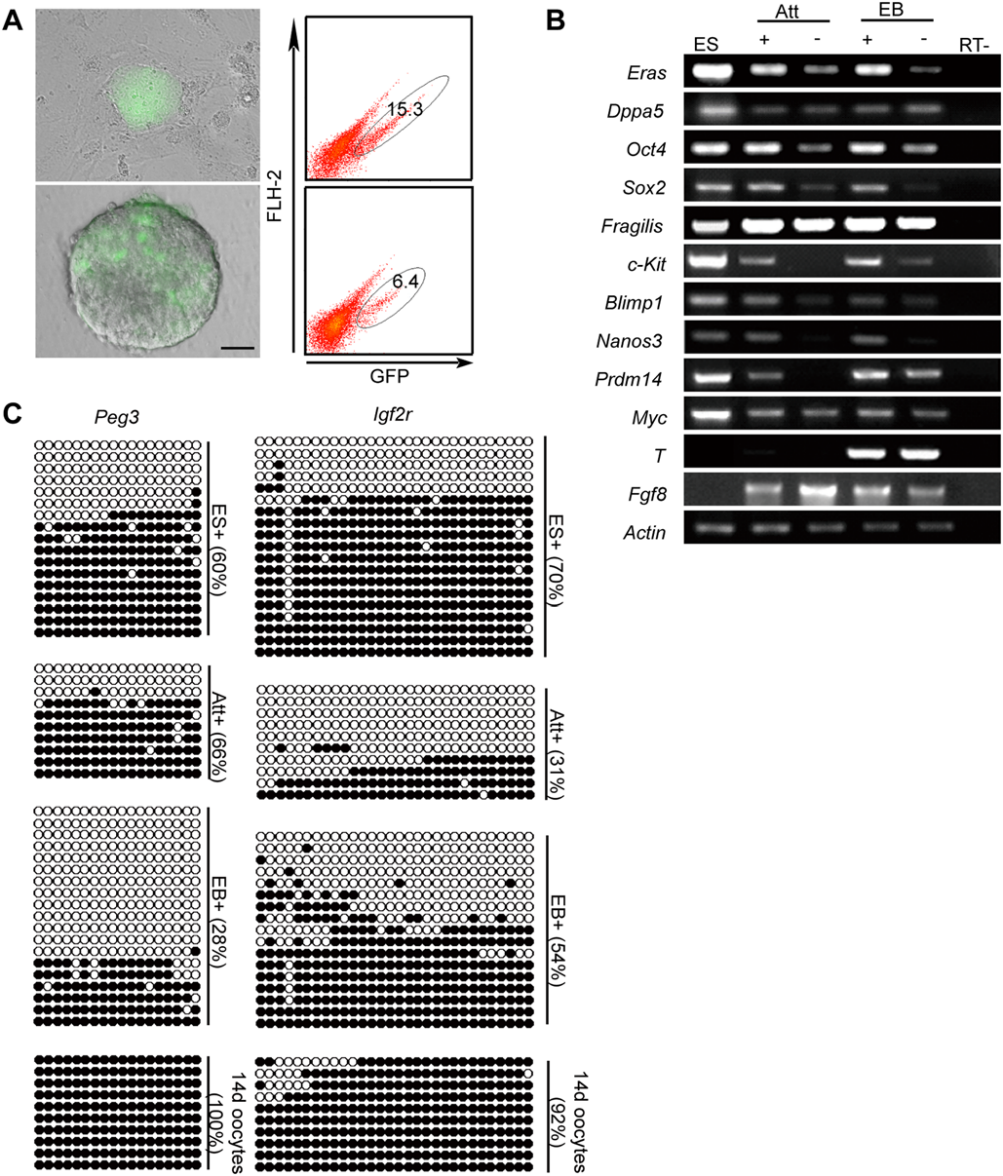
The derivation of PGCs from ES cells. (A) Merged view of the phase contrast images and the fluorescence images of *stella*-GFP expression (green) in day 7 of the attachment culture technique (upper panel) and day 4 of EB (lower panel); bar = 50 µm. GFP-positive cells in the attachment culture technique at day 7 accounted for 15% of all cells and formed clusters, whereas the cells in the EB method at day 4 accounted for 6% and were dispersed in the EB. (B) Gene-expression analysis of GFP-positive cells (+) and GFP-negative cells (−) in day 7 of the attachment culture technique (Att) and day 4 of the EB (EB). All of the detected genes are highly expressed during PGC specification, with the exception of *Eras* and *Dppa5*, which are repressed in PGCs [17]. (C) DNA methylation patterns for the differentially methylated regions (DMRs) of *Peg3* and for the DMRs of *Igf2r*. The oocytes on day 14 served as a control. The percentage of methylated CpG sites in the GFP-positive cells of the EScells, the day 7 attachment culture, and the day 4 EB (ES+, Att+, and EB+, respectively) were as indicated.

To investigate PGC derivation in *vitro*, we differentiated *stella*-GFP ES cells by implementing either the attachment culture technique or the EB method. Because *stella* is specifically expressed in PGCs during PGC specification *in vivo* [18], we wanted to determine whether the derived GFP-positive cells from a day 7 attachment culture (Att+) and a day 4 EB culture (EB+) contained PGCs (Fig. 2A). We first confirmed this after observing the strong expression of PGC markers, such as *Oct4*, *Sox2* and *Blimp1*, in Att+ and EB+ by RT-PCR (Fig. 2B), and the protein expression of Oct4, Mvh, c-Kit and SSEA1 by Immunocytochemical staining (Fig. S1). In addition, *Eras* and *Dappa5*, which are repressed in PGCs [17], were downregulated in Att+ and EB+, indicating the presence of PGCs in these samples (Fig. 2B). Moreover, we analyzed whether Att+ and EB+ were able to procure the erasure of parental imprints in a manner similar to that shown in PGCs *in vivo* [13]. The imprinted genes we chose were *Peg3* (5′ upstream region of the paternally expressed 3 gene), which is a paternally imprinted gene, and *Igf2r* (region 2 of the insulin-like growth factor 2 receptor gene), which is a maternally imprinted gene. Both imprints have been shown to exhibit early imprint erasure, with *Peg3* initiating the erasure earlier than *Igf2r in vivo* [13]. This enabled us to determine the change in early imprinting in derived cells and to follow the time course of imprinting erasure by detecting their methylation status. Thus, we examined the DNA methylation state of differentially methylated regions (DMRs) of *Peg3* and *Igf2r* (Fig. 2C). Upon comparison of Att+ to EB+, Att+ showed partial erasure of *Igf2r*, whereas EB+ displayed partial erasure of *Igf2r* and *Peg3* (Fig. 2C), demonstrating that both Att+ and EB+ contained PGCs. In addition, in EB+ the number of methylated CpG sites was significantly fewer in *Peg3* than in *Igf2r*, suggesting that *Peg3* was erased prior to *Igf2r*. The EB method, as opposed to the attachment culture technique, was able to recapitulate the erasure pattern of gene imprinting in the same sequential manner as that observed *in vivo*. Thus, PGCs were derived from ES cells by both the attachment culture technique and the EB method.

**Figure 3:**
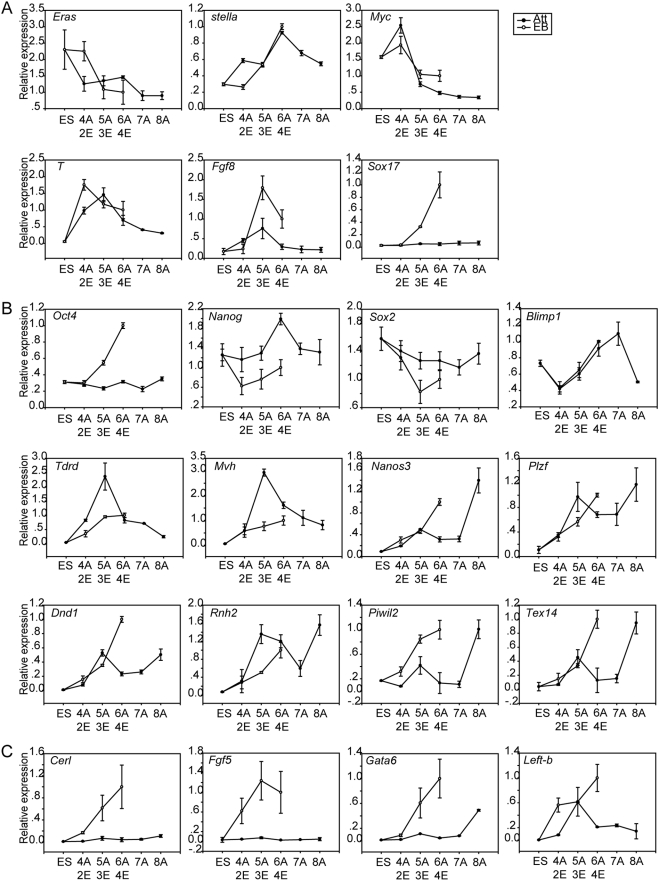
Gene-expression dynamics in GFP-positive cells from approximately days 4∼8 in the attachment culture technique (4A, 5A, 6A, 7A, 8A) and from days 2∼4 in the EB method (2E, 3E, 4E). (A) Genes differentially expressed between ES cells and PGCs. (B) PGC markers and germ cell markers, both expressed in ES cells and PGCs. (C) Epiblast markers. The relative expression of each gene in differentiated cultures was normalized by its expression in GFP-positive cells from day 4 EBs after normalization with *Gapdh*.

To explore the process of PGC derivation *in vitro* using these two methods, the gene expression dynamics of GFP-positive cells from days 4 to 8 in the attachment culture technique (Att+) and days 2 to 4 in the EB method (EB+) were analyzed by Quantitative RT-PCR (Q-PCR) (Fig. 3). First, the six genes that can distinguish ES cells and PGCs of different stages were analyzed. In both Att+ and EB+ cells, a decrease in the expression of *Eras* and an increase in the expression of *stella* further confirmed that PGCs were derived from ES cells using these two methods (Fig. 3A). The expression of *Myc* was also shown to decrease in both Att+ and EB+ (Fig. 3A) in a manner that is similar to its expression pattern *in vivo* [17]. Although both Att+ and EB+ showed increased expression of *T* and *Fgf8*, the expression level of *T* peaked on day 2, whereas *Fgf8* expression peaked on day 3 in the EB method (Fig. 3A). This suggested that its expression pattern resembled that of the *in vivo* process at roughly E7.25 when gene upregulation is followed by a subsequent downregulation and the change in the expression of *T* is earlier than that observed for *Fgf8* [17]. In comparison, the expression of both genes peaked on day 5 in the attachment culture technique. *Sox17* expression increased after day 3 in the EB method, mimicking the *in vivo* process [17], whereas it remained at low levels in the attachment culture technique (Fig. 3A). In addition, the expression pattern of some other PGC and germ cell markers, which are expressed in both PGCs and ES cells, such as *Oct4*, *Sox2* and *Blimp1*, fluctuated during PGC specification (Fig. 3B). Notably, in Att+ and EB+, the expression of *Blimp1* was downregulated followed by a upregulation, indicating the differentiation of ES cells followed by PGC specification (Fig. 3B). The expression of the epiblast markers, *Cerl*, *Fgf5*, *Gata6* and *Left-b*, increased significantly with the use of the EB method, whereas the expression of only *Left-b* increased clearly in the attachment culture technique (Fig. 3C), suggesting that an intermediate stage, perhaps an epiblast stage, existed during the process of PGC specification *in vitro* when the EB method was implemented. Thus, the process of PGC derivation was more faithfully recapitulated in the EB method.

### *In vitro* model of PGC specification

**Figure 4:**
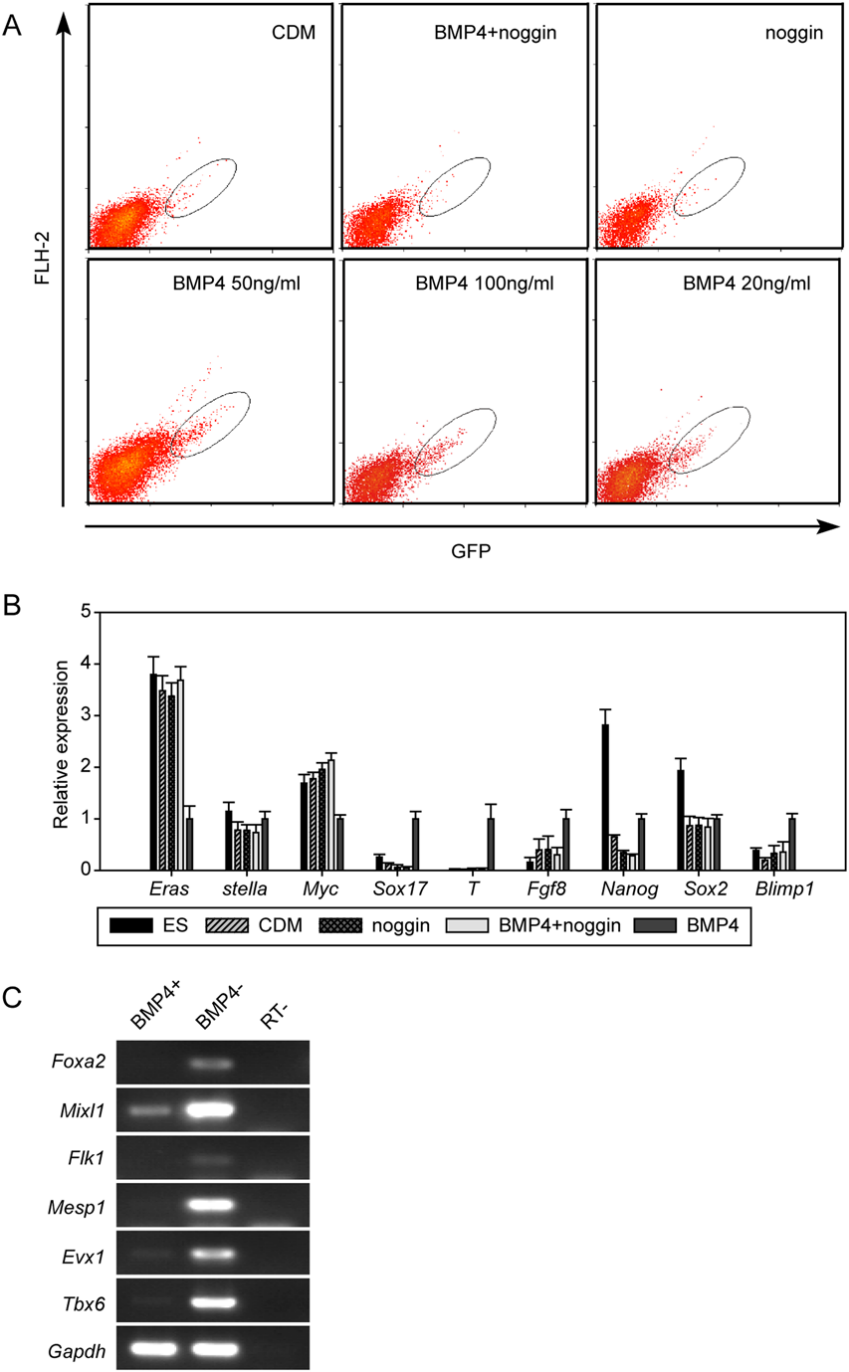
Induction of PGCs by BMP4 in the CDM. (A) Flow cytometric analysis of *stella*-GFP expression in day 4 EBs in the CDM or supplemented with BMP4, noggin or BMP4+ noggin. (B) Gene-expression analysis of PGC markers in GFP positive cells in (A). The relative expression of each gene in differentiated cultures was normalized by its expression in GFP-positive cells in the CDM with BMP4 after normalization with *Gapdh*. (C). Gene-expression analysis in GFP-positive (BMP4+) and GFP-negative (BMP4−) cells in day 4 EBs in the CDM with BMP4. *Foxa2* is a mesoendoderm marker, whereas *Mixl1*, *Flk1*, *Mesp1*, *Evx1* and *Tbx6* are mesoderm markers [24], [25]. *Gapdh* served as loading control.

To determine the signals that promote the derivation of PGCs, we attempted to develop an *in vitro* model of PGC specification. First, because the two subpopulations of *stella*-GFP ES cells were interchangeable regarding *stella* expression (Figs. 1B and C), we did not sort either of them to establish the model. Second, because the process of PGC derivation was more faithfully recapitulated in the EB method than in the attachment culture technique, we employed the EB method. Third, because unknown components coupled with the inherent variability in the quality of the serum are known to hamper the accuracy of the results [15], we decided to develop a completely chemically defined medium (CDM). In this respect, several basic media (including DMEM, DMEM/F12, Ham's F12, X-vivo, 1640, and IMDM) were tested, and we found that a combination of Ham's F12 with IMDM supported the survival of cells most effectively. Because the relative percentage of GFP-positive cells was extremely low in EBs formed in the CDM (0.78%) (Fig. 4A), the CDM model provided a strategy to study the signals that trigger PGC derivation.

Considering that BMP4, a mesoderm inducer, plays an important role in PGC generation *in vivo* [14], we applied BMP4 to our differentiation model. Interestingly, the percentage of GFP-positive cells was higher in day 4 EBs supplemented with BMP4 than in EBs with CDM alone, 2.96% and 0.78%, respectively (Fig. 4A). To detect whether the GFP-positive cells in the CDM supplemented with BMP4 included PGCs, the expression of the PGC-related genes, the 6 main genes and also *Nanog*, *Sox2* and *Blimp1*, was tested (Fig. 4B). As expected, in the presence of BMP4 the expression patterns of the PGC-related genes in GFP-positive cells were similar to the expression patterns in E7.25 PGCs (Fig. 4B), such as decreased expression of *Eras* and upregulated expression of *T* and *Fgf8*. In addition, the immunostaining results of the GFP-positive cells confirmed the expression of PGC markers, such as Oct4, Mvh, SSEA-1 and c-Kit (Fig. S2). The mesoderm induction effect of BMP4 was also confirmed (Fig. 4C). Thus, BMP4 was effective in promoting PGC specification in cells other than mesodermal cells derived from *stella*-GFP ES cells.

To further confirm the requirement of BMP4 in PGC specification, the BMP4 antagonist noggin was introduced to these samples. At day 2, noggin was added to the EB alone and to the CDM prior to the addition of BMP4. The percentage of GFP-positive cells in the noggin-alone (0.83%) and the noggin-BMP4 samples (0.67%) was similar to that of the CDM (0.78%), but lower than that of the CDM with BMP4 (2.96%) (Fig. 4A). In addition, all detected genes showed that the expression patterns of the GFP-positive cells in the presence of noggin were similar to those for the CDM (Fig. 4B). Thus, no PGCs differentiated from ES cells in a culture system that contained noggin. To determine whether PGC derivation was sensitive to the dose of BMP4, two other doses of BMP4 (20 and 100 ng/ml) were added to day 2 EBs in the CDM. The percentage of GFP-positive cells in the high dose (100 ng/ml) sample was 5.06%, whereas the percentage remained at roughly 3% in the medium dose (50 ng/ml, 2.96%) and low dose samples (20 ng/ml, 2.72%) (Fig. 4A). Interestingly, the expression patterns of the GFP-positive cells at all doses of BMP4 did not differ significantly (data not shown). These data further confirmed the role of BMP4 in inducing PGC differentiation from *stella*-GFP ES cells.

**Figure 5:**
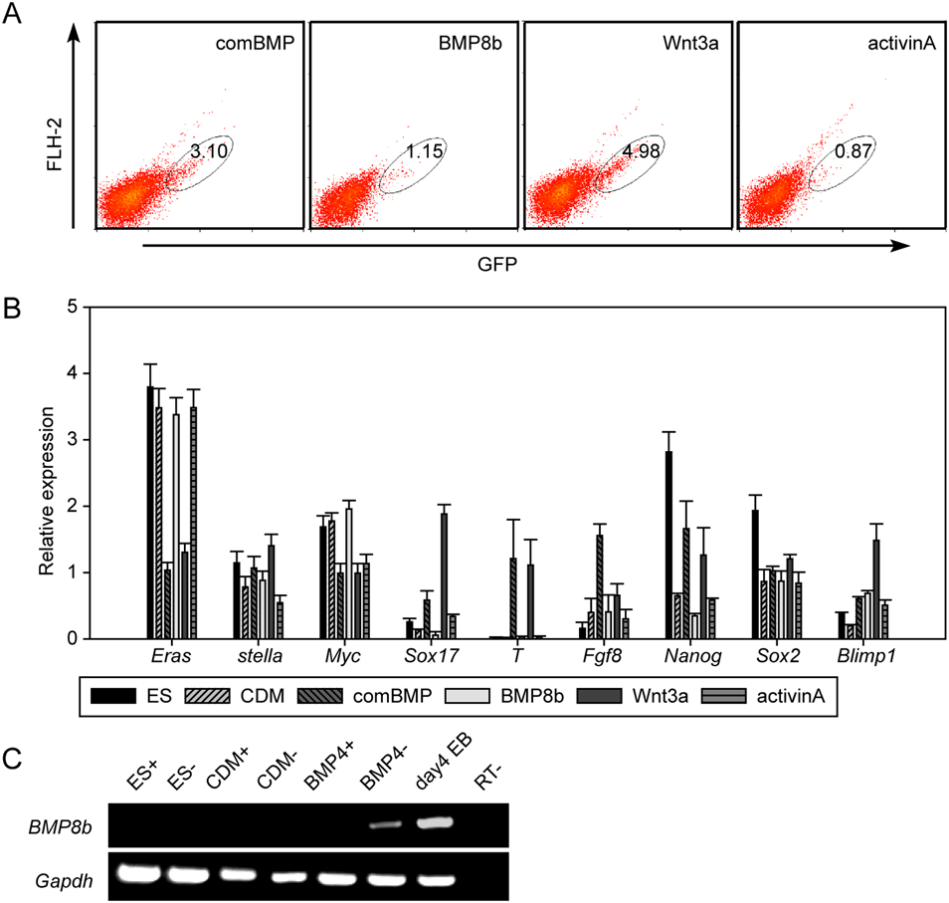
Screening the promotion effect of PGC specification by various factors. (A) Flow cytometric analysis of *stella*-GFP expression in day 4 EBs in the CDM supplemented with BMP2, BMP4 and BMP8b (comBMP), BMP8b, Wnt3a or activinA. (B) Gene-expression analysis of the PGC markers in GFP positive cells in (A). The relative expression of each gene in differentiated cultures was normalized by its expression in GFP-positive cells in the CDM with BMP4 after normalization with *Gapdh*. (C). The expression of BMP8b in GFP-positive or GFP-negative cells in ES (ES+, ES−), in day 4 EBs in the CDM (CDM+, CDM−), in day 4 EBs in the CDM with 50 ng/ml BMP4 (BMP4+, BMP4−) and in day 4 EBs in serum (day 4 EB). The day 4 EBs in serum served as a positive control.

Because another two BMP proteins, BMP8b and BMP2, also promote PGC specification by assisting BMP4 *in vivo* [20]–[22], BMP8b and BMP2 were used to supplement the EBs in the CDM containing BMP4 (comBMP) at day 2. The percentage of GFP-positive cells by adding comBMP (3.1%) and the expression patterns of these cells were similar to that observed for BMP4 alone (Fig. 5A,B). Because BMP8b shows no additive effect with either BMP4 or BMP2 *in vivo* [20]–[22], suggesting different roles of BMP8b in PGC specification, BMP8b alone was added at day 2 to the EBs in the CDM. Both the overall percentage of GFP-positive cells (1.15%) and the gene expression pattern of these cells were similar to that in the CDM, indicating that BMP8b alone was not sufficient to promote the generation of PGCs (Figs. 5A and B). Furthermore, because the absence of BMP8b is known to result in an absence of PGC generation i*n vivo* [20], [21], we examined whether BMP4 induced the expression of BMP8b in the CDM. The expression of BMP8b was detected in the GFP-negative cells in day 4 EBs in the CDM with BMP4 but not in the GFP-positive cells, the ES cells, or the day 4 EBs in the CDM (Fig. 5C). These results demonstrated that no synergic effect was detected by adding BMP8b or BMP2 with BMP4, while BMP4 can stimulate the expression of BMP8b in non-PGC cells in the CDM.

Because BMP4 is also a member of a group of proteins known as mesoderm inductors [23], Wnt3a, another factor promoting mesoderm lineage[24], was tested. As expected, Wnt3a had a positive effect on PGC derivation similar to that observed for BMP4 (Figs. 5A and B). Subsequently, activinA, which induces the generation of both the mesoderm and the endoderm [25], was shown to act in a similar fashion to BMP8b (Figs. 5A and B) with no induction of PGC specification. Thus, Wnt3a stimulated PGC specification in a manner similar to BMP4, whereas BMP8b and activinA failed to stimulate PGC specification.

## Discussion

In this study, we successfully derived PGCs from mouse ES cells using *stella* as a selective marker. Interestingly, our data demonstrated that although ES cells were heterogeneous, they may not contain cells that are equivalent to PGC precursors or PGCs prior to E7.75. In addition, we found that the process of PGC differentiation from ES cells underwent complicated changes in the patterns of gene expression and methylation status, therefore mimicking the *in vivo* PGC generation process. Moreover, this process was more faithfully recapitulated when implementing the EB method than when using the attachment culture technique, suggesting a significant influence of the precise method on the ES cell differentiation process. Furthermore, to our knowledge, this is the first report demonstrated that PGCs were capable of being derived in a completely CDM, and our results showed that BMP4 and Wnt3a promoted PGC derivation from ES cells, whereas BMP8b and activinA were unable to promote PGC derivation.

Our data showed that ES cells were heterogeneous regarding their ability to express *stella*-GFP and that the two populations were capable of generating one another. This phenomenon is similar to that reported for *Rex1* in ES cells [26] and thus serves to confirm their reported heterogeneity. We also found that the two subpopulations of ES cells not only differed in the gene expression pattern (Figs. 1D and E), but also showed different DNA methylation patterns in imprinted genes (Figs. 2C and S3), consistent with previous studies indicating that ES cells are epigenetically unstable [27]. However, neither of the subpopulations possessed expression patterns similar to PGC precursors or PGCs prior to E7.75 (Fig. 1E, Table 1). Recently, Hayashi *et al.* also reported on the heterogeneity of ES cells regarding the expression of *stella* [28]. Through single cell Q-PCR analysis, they found that Stella-positive ES cells are closely related to the inner cell mass and not related to the epiblast or PGCs, whereas Stella-negative ES cells are more similar to the epiblast cells [28]. Taken together, these results suggest that ES may not contain either PGCs or PGC precursors.

By following these detailed differentiation dynamics, we have found that, in comparison to the attachment culture technique, the process of PGC specification was more faithfully recapitulated using the EB method. Our results indicated that the imprinted genes were able to procure the erasure in *stella*-positive cells when the EB method (EB+) was used, whereas only *Igf2r* was able to procure the erasure when the attachment culture (Att+) technique was implemented (Fig. 2C). The sequential erasure of *Peg3* followed by *Igf2r* was detected in EB+ but not in Att+ (Fig. 2C). In addition, our results showed that the gene expression dynamics of specific PGC markers, such as *T*, *Fgf8* and *Sox17* in the EB method, resembled the expression of *in vivo* markers more closely than the markers observed using the attachment culture technique (Fig. 3A). Finally, our results indicated that all of the levels of the detected epiblast genes were remarkably higher in the EB+, whereas only the level of *Left-b* was higher in Att+, indicating the presence of an epiblast stage in the PGC specification process when analyzed using the EB method. This observation was similar to that shown for the PGCs derived from proximal epiblasts *in vivo* [14]. Thus, the EB method presented a process of PGC specification that more closely mimicked the *in vivo* process.

Our data demonstrated that BMP4 was sufficient to promote PGC specification in the CDM. Initially, we discovered that BMP4 functioned as a soluble protein. In contrast to our findings, Toyooka *et al.* indicated that the direct addition of BMP4 to the medium, or simply co-culturing cells with BMP4-producing cells as feeders, does not necessarily stimulate PGC production [7]. A possible reason for the latter observation is that the serum in their culture medium includes factors that perform functions similar to BMP4 or interfere with BMP4, while our CDM culture eliminated this complicated effect by providing a far superior strategy to study the signals in PGC specification. In addition, our data suggest that the dose of BMP4 regulates the efficiency of PGC specification. Upon increasing the dose of BMP4 from 50 to 100 ng/ml, the percentage of GFP-positive cells increased (Fig. 4A) in a manner that was consistent with previous studies demonstrating that mice heterozygous for the BMP4-null generate fewer PGCs than wild-type mice [14]. This aforementioned result was also consistent with a previous study demonstrating that the PGC number is regulated by BMP signaling in an organ culture [29]. Moreover, it is possible that BMP4 triggers PGC derivation by providing a favorable microenvironment in our model. BMP4 induced the expression of BMP8b in GFP-negative cells in the CDM, which are the nearby cells of our derived PGCs in the EBs (Fig. 5C), consistent with previous findings that BMP8b expressed in the extraembryonic ectoderm is necessary for PGC specification from an epiblast precursor [20]. However, because BMP8b alone is not sufficient to promote PGC derivation (Figs. 5A and B), BMP4 must have an additional effect on this process. Interestingly, BMP4 can induce the formation of the extraembryonic mesoderm [30], [31] where PGCs form a cluster and undergo further development. Therefore, BMP4 may establish a proper microenvironment for PGC specification and development. Thus, the functions performed by BMP4 in PGC specification were represented in our *in vitro* model.

**Figure 6:**
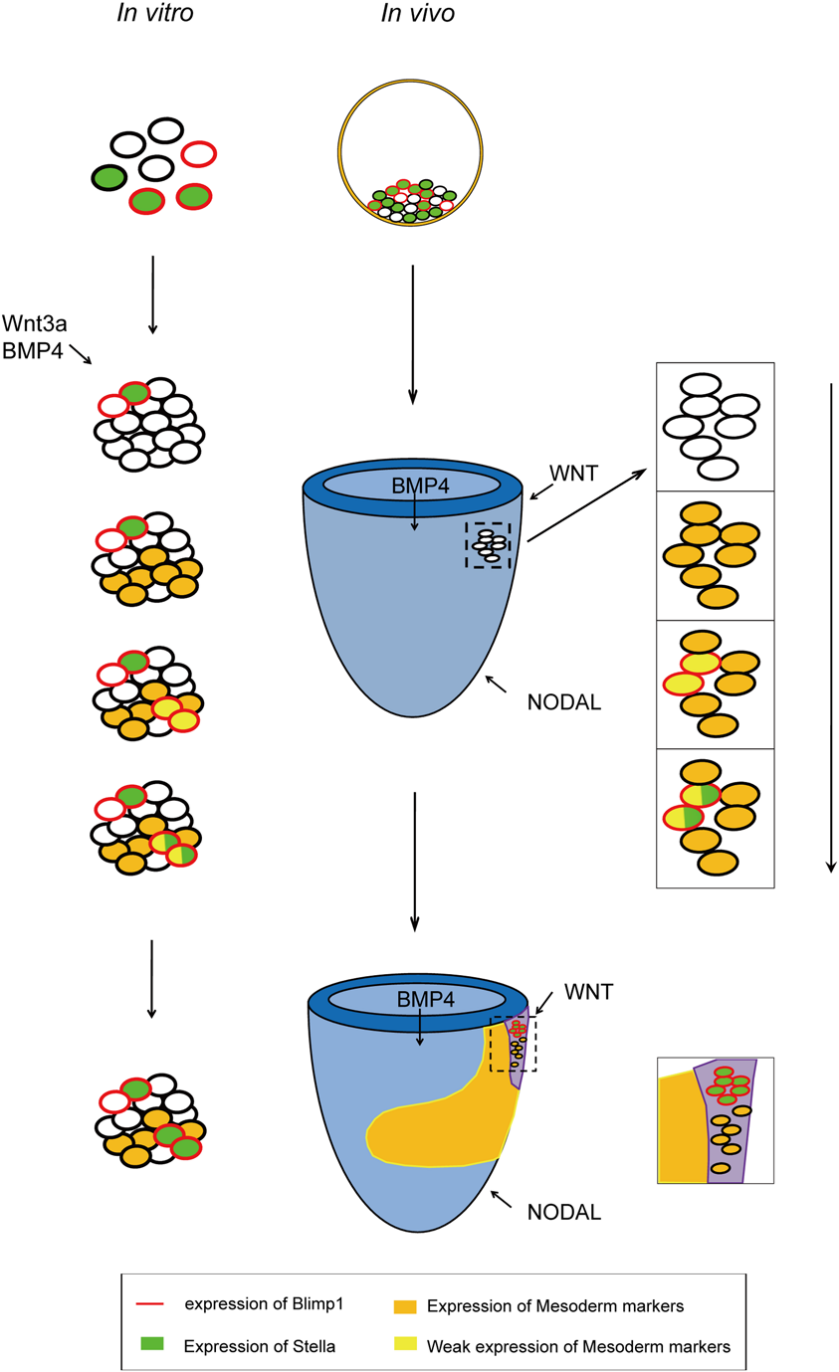
A potential model of PGC specification. Some of the cells in the ICM (inner cell mass)/ES cells were Stella and/or Blimp1 positive cells. with the development of the embryo, the expression of Blimp1 was depressed in these cells with the help of signals from neighboring cells. Later, induced by WNT and/or BMP signaling, a subpopulation of these cells with the expression of mesoderm markers emerged, some of which acquired the expression of Blimp1. Then, Blimp1 functions to repress the expression of somatic markers that were normally down-regulated in PGCs [34]. Subsequently, cells with the expression of both Blimp1 and Stella were fated to germ-line development.

Our results suggested that the germ cell fate was related to the fate of the mesoderm. Here, we found that a majority of the detected mesoderm markers were confirmed in the GFP-positive cells of the CDM in the presence of BMP4, which is consistent with an *in vivo* study demonstrating that mesoderm markers are expressed in some nascent PGCs while being repressed in PGCs [18]. However, Wnt3a and activinA, both of which induce mesoderm production *in vitro* [23]–[25], were shown to have different effects on PGC specification (Fig. 5A,B). Recently, a study by Gadue *et al.* revealed that upon direct addition of the two factors individually, Wnt3a is responsible for the induction of a population of cells with Foxa2^low^T^+^, which are cells in the posterior part of the primitive streak, whereas activinA induces cells with Foxa2^high^T^+^, which are cells of the anterior part of the primitive streak [32]. Thus, the promotion effect of Wnt3a and negative effect of activinA in PGC specification suggest that either PGCs originate from the posterior part of the primitive streak or the germ cell fate is imposed on these cells in the primitive streak. Together, it is possible that, during gastulation the precursors of putative PGCs and nascent mesoderm cells, expressing mesoderm markers, such as *Evx1*, *Tbx1* and *Mesp1*, were segregated from other somatic cells with the induction of BMP4 and/or Wnt3a. Soon after this segregation, the upregulation of *Blimp1* in some of these precursors repressed the expression of mesoderm markers and finally the Blimp1-positive cells destined for a germ cell fate. (Fig. 6). Hence, our model provides a novel method to screen for factors or small molecules that may be involved in PGC specification.

## Materials and Methods

### ES Cell Maintenance

The *stella*-GFP ES cells (a gift from Prof. M. Azim Surani, also described as *stella*-GFP BAC ES cells in their publication [19]) were maintained in an ES medium that consisted of DMEM/F12 (Invitrogen) supplemented with 15% fetal calf serum (Hyclone), 1 mM glutamine (Invitrogen), 100 U/ml penicillin/streptomycin (Sigma), 0.1 mM β-mercaptoethanol (Sigma), and 1,000 U/ml LIF (Sigma) on mitomycin C-treated mouse embryonic fibroblasts.

### ES Cell Differentiation

ES cells were differentiated in either a serum-containing medium (ES medium without LIF) or a serum-free medium, the components of which were described previously by Gadue *et al.* [32], containing 75% Iscove's modified Dulbecco's medium (Invitrogen), 25% Ham's F12 medium (Invitrogen) with 0.5-fold of both N2 and B27 (without retinoic acid) (Invitrogen), 0.05% BSA (Sigma), 2 mM glutamax (Invitrogen), 0.5 mM ascorbic acid (Sigma) and 4.5×10^−4^ M 1-thioglycerol (Sigma). The attachment culture technique and EB method were performed as previously described [6], [32]. Briefly, after the ES cells were trypsinized, disassociated ES cells were plated on gelatin-treated plates for 40 min to remove the feeder cells. These cells were subsequently filtered through a 75-µm cell strainer. The ES cells were seeded at a density of 1 to 2.5×10^4^ cells per cm^2^ in a serum-containing medium in the attachment culture technique at a density of 0.5×10^5^ cells/ml in serum-containing medium or at 1.5×10^5^ cells/ml in a serum-free medium in the EB culture. After 48 h, the EBs were dissociated, filtered, and seeded as primary EBs to generate secondary EBs. The secondary EBs were cultured in either a serum-containing medium or a serum-free medium supplemented with BMP4, noggin, BMP2, BMP8b, Wnt3a or activinA, as indicated.

### Flow cytometry

Cells were dissociated in trypsin-EDTA and resuspended in the medium used previously. The cells were then placed in a MoFlo High-Performance Cell Sorter (Dako Cytomation, Glostrup, Denmark) using Summit 4.0 Software (Dako Cytomation) for analysis and sorting.

### Reverse Transcription Polymerase Chain Reaction (RT-PCR) analysis and Q-PCR analysis

The total RNA was extracted using an RNeasy Micro Kit (Qiagen). The RNA was then reverse-transcribed into cDNA using Sensiscript RT Kits (Qiagen). PCR was performed with Ex Taq polymerase (Takara) in a PCR buffer. The cycle conditions were as follows: 94°C for 5 min followed by 28–30 cycles of a 94°C denaturation period for 40 sec, a 56–60°C annealing period for 40 sec, and a 72°C elongation period for 40 sec, with a final elongation period at 72°C for 10 min. The primers used are listed in Table S1.

Q-PCR analysis was performed on an ABI PRISM 7300 Sequence Detection System using the SYBR Green PCR Master Mix (TOYOBO). The PCR consisted of 12.5 µl of SYBR Green PCR Master Mix, 1 µl of 10 mM forward and reverse primers, 10.5 µl water, and 1 µl template cDNA in a total volume of 25 µl. Cycling was performed using the default conditions of ABI 7300 SDS Software 1.3.1: 2 min at 95°C, followed by 30–35 cycles of 15 sec at 95°C and 1 min at 60°C. The relative expression of each gene was first normalized against *Gapdh*. The expression results for each gene were subsequently normalized by the expression with respect to the selected sample in each group, as indicated in each figure. The primers used for Q-PCR are shown in Table S2.

### DNA Methylation Analysis

Genomic DNA was extracted using the DNeasy kit (Qiagen). The DNA was then treated with a sodium bisulfite solution, as described previously [33]. Differentially methylated regions (DMRs) of *Igf2r* or *Peg3* were amplified by Ex-Taq DNA polymerase (TaKaRa) via nested PCR. The conditions for the first round of cycling were as follows: 94°C for 5 min followed by 35 cycles of a 94°C denaturation period for 30 sec, a 55°C annealing period for 30 sec, and a 72°C elongation period for 60 sec, with a final elongation period at 72°C for 10 min. The second round of PCR cycling was as follows: 94°C for 5 min followed by 35 cycles of a 94°C denaturation period for 30 sec, a 58°C annealing period for 30 sec, and a 72°C elongation period for 60 sec, with a final elongation period at 72°C for 10 min. The PCR primers are listed in Table S3. Amplified fragments were cloned into the plasmid vector pGEM-T Easy (Promega), and 10 samples in each experiment were sequenced using an ABI PRISM 3100 Genetic Analyzer (Applied Biosystems, Foster City, CA).

### Immunocytochemical analysis

The cells were treated as described previously [12]. Briefly, the cells were fixed in 4% paraformaldehyde and blocked with 10% normal goat serum and 0.2% Triton X-100 for 60 min at room temperature. The cells were then incubated overnight at 4°C with the primary antibody to Oct4 (rabbit polyclonal IgG, Abcam), Mvh (rabbit polyclonal IgG, a kind gift from Dr. Toshiaki Noce), SSEA-1 (mouse monoclonal IgG, Chemicon) or c-kit (rabbit polyclonal IgG, Chemicon). Further incubation with anti-rabbit tetramethylrhodamine isothiocyanate (TRITC)-conjugated IgG or anti-mouse TRITC (both from Santa Cruz) was performed for 45 min at room temperature. The cells with only secondary antibody staining served as negative controls. The nuclei were detected by DAPI (Roche) staining. The images were obtained with an Olympus phase contrast microscope (IX-71; Olympus).

### Statistical analysis

All data presented are representative of at least three independent experiments unless indicated otherwise. The results are expressed as the mean±s.e.m. of at least three independent experiments. Statistical analysis was performed using one-way ANOVA, followed by the SNQ test if necessary. The data collected from Quantitative RT-PCR were analyzed with the original data normalized with *Gapdh*. The statistical significance was inferred at * P<0.05 and ** P<0.01.

## Supporting Information

Figure S1 Immunostaining of PGC markers, Oct4, Mvh, SSEA-1 and c-Kit in differentiated cells derived from ES cells by attachment culture (four upper panels) or the EB method (cells were dissociated from EBs before staining, four lower panels). Nuclei were visualized by Dapi. Bar = 50 µm.
(1.87 MB TIF)

Figure S2 Immunostaining of PGC markers Oct4, Mvh, SSEA-1 and c-kit in sorted GFP positive cells in day 4 EB in CDM with BMP4. Nuclei were visualized by Dapi. Bar = 50 µm
(0.24 MB TIF)

Figure S3 DNA methylation patterns of Peg3 differentially methylated regions (DMRs) and Igf2r DMRs. The percentage of methylated CpG sites in GFP-negative cells in ES cells, day 7 attachment culture and day 4 EB (ES−, Att−, EB−, respectively) were as indicated.
(0.49 MB TIF)

Table S1 (0.04 MB DOC)

Table S2 (0.05 MB DOC)

Table S3 (0.03 MB DOC)
