# Supplementary figures and images for: Primordial Germ Cell Specification from Embryonic Stem Cells
Source: PLoS One. 2008 Dec 24;3(12):e4013. doi: 10.1371/journal.pone.0004013 (PMC2602984; doi:10.1371/journal.pone.0004013)

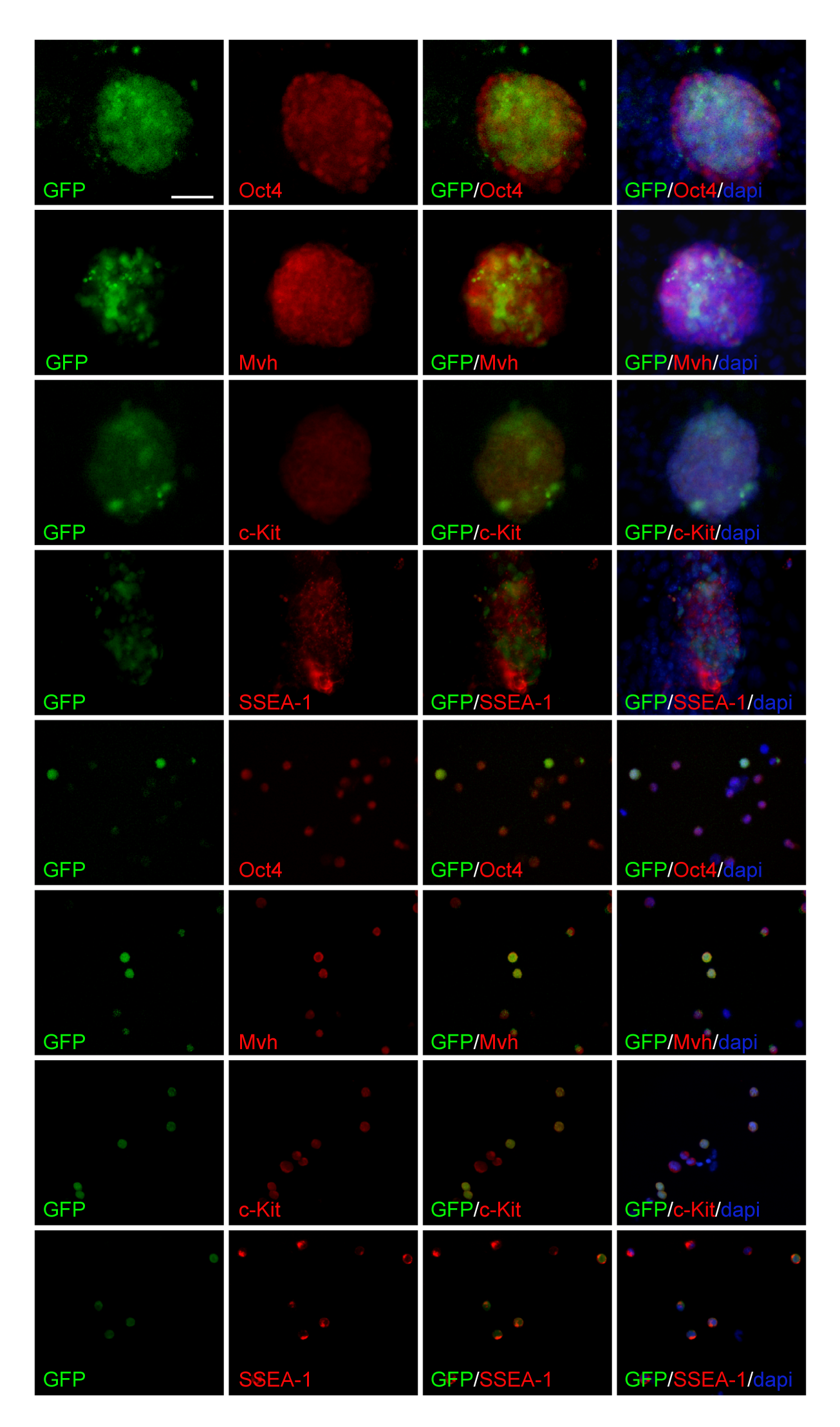

Supplement: Figure S1 — Immunostaining of PGC markers, Oct4, Mvh, SSEA-1 and c-Kit in differentiated cells derived from ES cells by attachment culture (four upper panels) or the EB method (cells were dissociated from EBs before staining, four lower panels). Nuclei were visualized by Dapi. Bar = 50 µm. (1.87 MB TIF) [file pone.0004013.s001.tif]

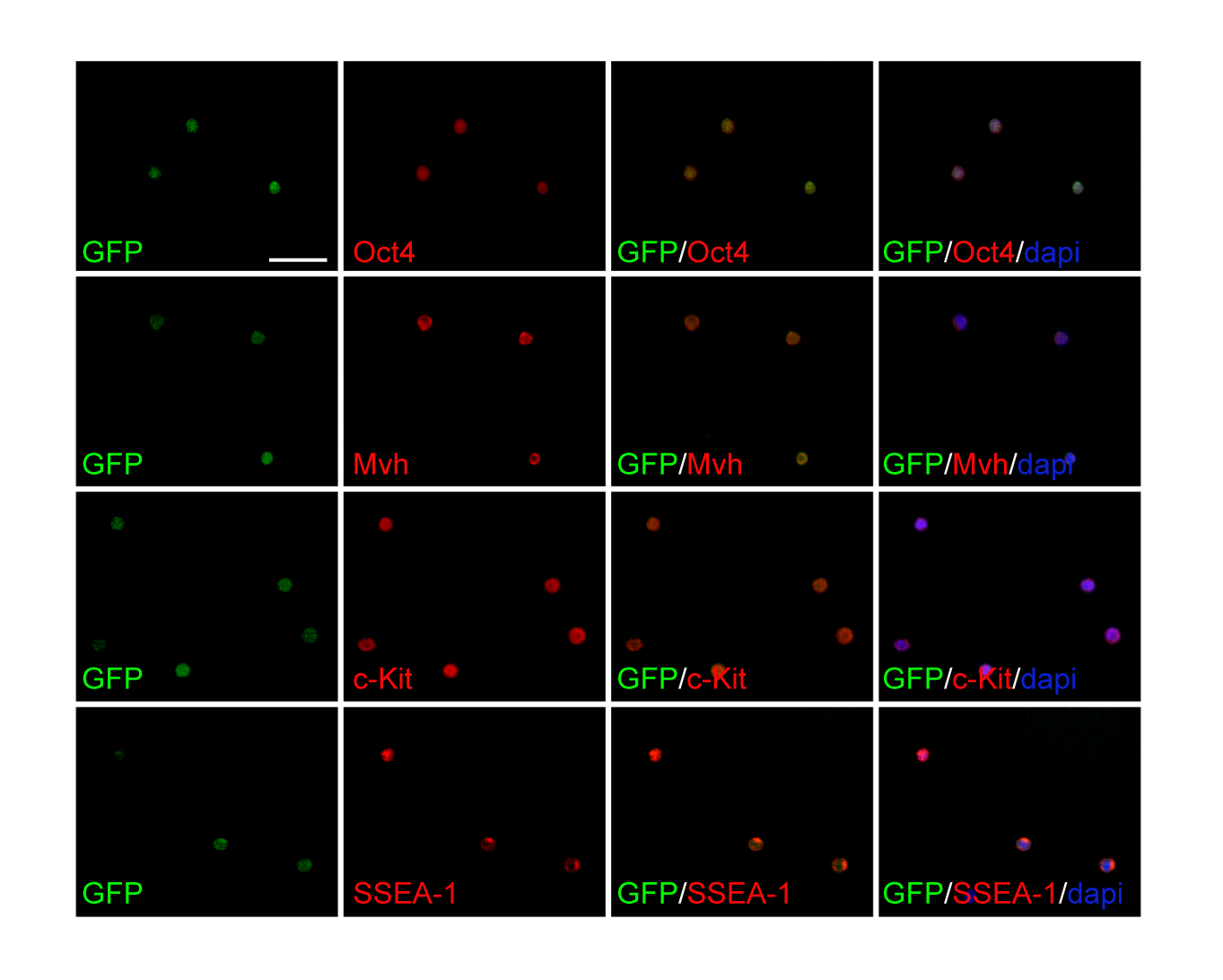

Supplement: Figure S2 — Immunostaining of PGC markers Oct4, Mvh, SSEA-1 and c-kit in sorted GFP positive cells in day 4 EB in CDM with BMP4. Nuclei were visualized by Dapi. Bar = 50 µm (0.24 MB TIF) [file pone.0004013.s002.tif]

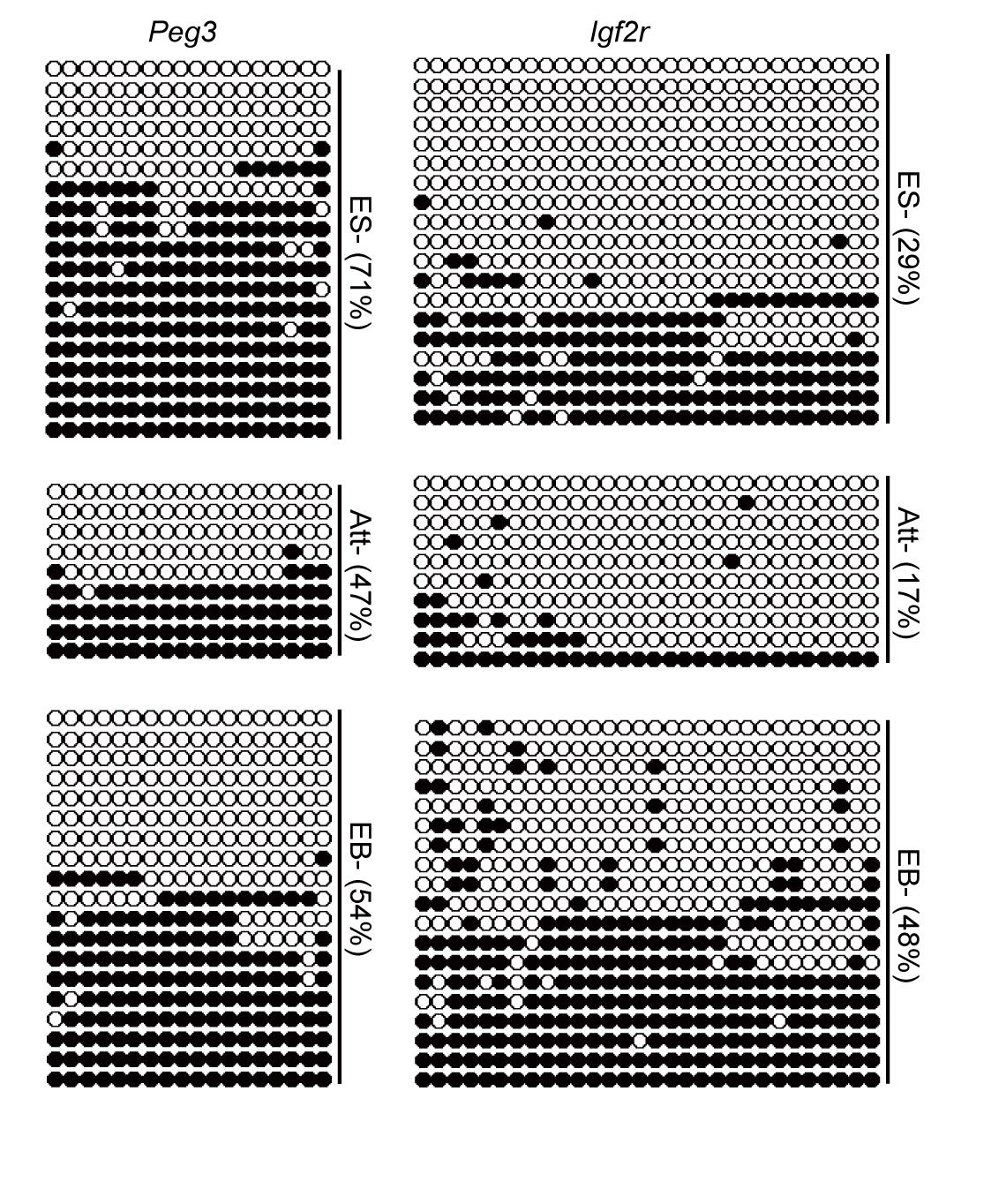

Supplement: Figure S3 — DNA methylation patterns of Peg3 differentially methylated regions (DMRs) and Igf2r DMRs. The percentage of methylated CpG sites in GFP-negative cells in ES cells, day 7 attachment culture and day 4 EB (ES−, Att−, EB−, respectively) were as indicated. (0.49 MB TIF) [file pone.0004013.s003.tif]
