# Supplementary material for: Primordial Germ Cell Specification from Embryonic Stem Cells
Source: PLoS One. 2008 Dec 24;3(12):e4013. doi: 10.1371/journal.pone.0004013 (PMC2602984; doi:10.1371/journal.pone.0004013)
Supplement: Table S1 — (0.04 MB DOC) [file pone.0004013.s004.doc]

**Table S1 Primers for RT-PCR**

| Gene | Primer1 | Primer2 | Size (bp) |
| --- | --- | --- | --- |
| *Oct4* | AAGCCCTGCAGAAGGAGCTAGAAC | GGGGCAGAGGAAAGGATACAG | 576 |
| *Fragilis* | TGCCTTTGCTCCGCACCAT | GGGTGAAGCACTTCAGGACC | 469 |
| *Dppa5* | AAGGAGTGCTGAAGCTGGAGG | CAGCTTAACCTGCATCCAGGTC | 110 |
| *Stella* | AGGCTCGAAGGAAATGAGTTTG | TCCTAATTCTTCCCGATTTTCG | 118 |
| *Nanos3* | CACTACGGCCTAGGAGCTTGG | TGATCGCTGACAAGACTGTGGC | 127 |
| *Blimp1* | TGGCGGATCTATTCCAGA | CCCGGATAGGATAAACCAC | 266 |
| *Sox2* | CATGAGAGCAAGTACTGGCAAG | CCAACGATATCAACCTGCATGG | 127 |
| *Prdm14* | ACAGCCAAGCAATTTGCACTAC | TTACCTGGCATTTTCATTGCTC | 126 |
| *c-kit* | CTGCTTGGCGCATGCACGG | CCGGCATCCCTGGGTAGGG | 655 |
| *Foxa2* | TGGTCACTGGGGACAAGGGAA | GCAACAACAGCAATAGAGAAC | 289 |
| *Mixl1* | GCACGTCGTTCAGCTCGGAGCAGC | AGTCATGCTGGGATCCGGAACGTGG | 305 |
| *Flk-1* | CACCTGGCACTCTCCACCTTC | GATTTCATCCCACTACCGAAAG | 239 |
| *Evx1* | CAACCTAGTAGCTCAGACACCGAA | CGGTCTTGAAACGTAGTTCTCCCT | 309 |
| *Tbx6* | CCTTCCGATTTCCTGAGA-CCACAT | AGGTCCAGAAATGCAGCTGAGTAG | 455 |
| *Mesp1* | TCCCTCATCTCCGCTCTTCAGC | GGTTGGAATGGTACAGTCTGGATGAG | 253 |
| *BMP8b* | CCTGGCTCTGTGCGTGTT | CTGGGTTAGGTCAAAGTGGAAT | 359 |
| *T* | ATGCCAAAGAAAGAAACGAC | AGAGGCTGTAGAACATGATT | 834 |
| *Fgf8* | GCTGTTGCACTTGCTGGTT | GCCTTTGCCGTTGCTCTT | 499 |
| *Hoxa1* | GTGACTAGTCTTCTGCATGTCG | TCTGCTCTGGACCACATCACTC | 155 |
| *Eras* | TGGGCGTCTTTGCTCTTG | TCGGGTCTTCTTGCTTGATT | 293 |
| *Gapdh* | GTCATTGAGAGCAATGCCAG | GTGTTCCTACCCCCAATGTG | 215 |
| *Actin* | TCGTGGGCCGCTCTAGGCAC | TGGCCTTAGGGTTCAGGGGG | 243 |
