# Supplementary material for: Primordial Germ Cell Specification from Embryonic Stem Cells
Source: PLoS One. 2008 Dec 24;3(12):e4013. doi: 10.1371/journal.pone.0004013 (PMC2602984; doi:10.1371/journal.pone.0004013)
Supplement: Table S2 — (0.05 MB DOC) [file pone.0004013.s005.doc]

**Table S2 Primers for Quantitative RT-PCR**

| Gene | Primer1 | Primer2 | Size (bp) |
| --- | --- | --- | --- |
| *Myc* | AAGGAGAACGGTTCCTTCTGAC | GCTGAAGCTTACAGTCCCAAAG | 106 |
| *Sox17* | TTCTGTACACTTTAATGAGGCTGTTC | TTGTGGGAAGTGGGATCAAG | 134 |
| *T* | ATCAGAGTCCTTTGCTAGGTAG | GTTACAATCTTCTGGCTATGC | 194 |
| *Eras* | GTAGCTGTGGCTGCTCTGTAG | GATGTCTGTGGTAACTTGGTCG | 121 |
| *Fgf8* | CATGGCCTTTACCCGCAAG | CGGGTAGTTGAGGAACTCGAAG | 143 |
| *Nanog* | CTTTCACCTATTAAGGTGCTTGC | TGGCATCGGTTCATCATGGTAC | 112 |
| *Sox2* | CATGAGAGCAAGTACTGGCAAG | CCAACGATATCAACCTGCATGG | 127 |
| *Blimp1* | AGCATGACCTGACATTGACACC | CTCAACACTCTCATGTAAGAGGC | 162 |
| *Tex14* | ACTAACCTCGTGTGGAATAGGA | GGACAAACATACAGCCATCAGTT | 168 |
| *Mvh* | GCTTCATCAGATATTGGCGAGT | GCTTGGAAAACCCTCTGCTT | 193 |
| *Nanos3* | CACTACGGCCTAGGAGCTTGG | TGATCGCTGACAAGACTGTGGC | 127 |
| *Dnd1* | GAGGTGTATATCGGACGACTTCC | GGTTCAAACCACTGAAGGTCATC | 118 |
| *Piwil2* | GTTCCTCTTTGCCTGATCCTTC | AGAGACCTCAGAACTACCTCTTC | 107 |
| *Rnh2* | AAAAGCTACTCATGTGTTGGCA | ACCAAAGAGGCTGTTTCATTGA | 109 |
| *Plzf* | CCCAGTTCTCAAAGGAGGATG | TTCCCACACAGCAGACAGAAG | 88 |
| *Cerl* | ATCACCTCTACAGGAGGAAGC | GGTCTCCCAGTGTACTTCGTG | 115 |
| *Gata6* | GGCAGTGTGAGTGGAGGTG | TGGTACGTTCCGTTCAGCG | 101 |
| *Fgf5* | AAGTAGCGCGACGTTTTCTTC | CTGGAAACTGCTATGTTCCGAG | 94 |
| *Left-b* | GTGAATCTGACCCGAAGCAAT | CACGTCGGCTCCAATCTCA | 111 |
| *c-Kit* | CAGTTACCGCGCTCTGTTTG | GCCCCTTAAGTACCTGACATCC | 101 |
| *stella* | AGGCTCGAAGGAAATGAGTTTG | TCCTAATTCTTCCCGATTTTCG | 124 |
| *Oct4* | GATGCTGTGAGCCAAGGCAAG | GGCTCCTGATCAACAGCATCAC | 145 |
| *Tdrd1* | ACAGACCAACTCCAGGCGAT | GCCTCTTTTGTCCAATGCTTG | 110 |
| *Gapdh* | ATGAATACGGCTACAGCAACAGG | CTCTTGCTCAGTGTCCTTGCTG | 105 |
