# Supplementary material for: Primordial Germ Cell Specification from Embryonic Stem Cells
Source: PLoS One. 2008 Dec 24;3(12):e4013. doi: 10.1371/journal.pone.0004013 (PMC2602984; doi:10.1371/journal.pone.0004013)
Supplement: Table S3 — (0.03 MB DOC) [file pone.0004013.s006.doc]

**Table S3 Primers for nested PCR**

| Gene | Primer1 | Primer2 | Size (bp) |
| --- | --- | --- | --- |
| *Igf2r* 1^st^ | CACC TTCATTTACA TAACCAATAA | TTTAGAGGATTTTAGTATAATTTTAA | 666 |
| *Igf2r* 2^nd^ | CACTTTTAA ACTTACCTCT CTTAC | GAGGTTAAGGGTGAAAAGTTGTAT | 501 |
| *Peg3* 1^st^ | TTGTTGATGTTAATTTTGTGTTTTGGTG | TCAACCTTATCAATTACCCTTAAAAACC | 489 |
| *Peg3* 2^nd^ | TTTTGTAGAGGATTTTGATAAGGAGGTG | CCCCAAACACCATCTAAACTCTACAAAC | 288 |
